# Clinical Translation of Fisetin for Age-Related Diseases: Current Evidence and Future Opportunities

**DOI:** 10.3390/nu18182999

**Published:** 2026-09-14

**Authors:** Carolina Sandoval-Caballero, Zander Roemer, Catherine M. Kotz, Michael A. Puskarich, Vijayakumar Mavanji, Elizabeth L. Schmidt, Shalamar D. Sibley

**Affiliations:** 1 Department of Integrative Biology and Physiology, University of Minnesota, Minneapolis, MN 55455, USA; clsandov@umn.edu (C.S.-C.); roeme047@umn.edu (Z.R.); kotzx004@umn.edu (C.M.K.); 2 Geriatric Research, Education and Clinical Center (GRECC), Minneapolis VA Health Care Center, Minneapolis, MN 55417, USA; 3 Department of Emergency Medicine, University of Minnesota, Minneapolis, MN 55364, USA; mike-em@umn.edu; 4 Department of Emergency Medicine, Hennepin Healthcare, Minneapolis, MN 55364, USA; 5 Research Service, Minneapolis VA Health Care System, Minneapolis, MN 55417, USA; vijaya.mavanji@va.gov; 6 Department of Biochemistry, Molecular Biology, and Biophysics, University of Minnesota, Minneapolis, MN 55455, USA; thom4573@umn.edu; 7 Masonic Institute on the Biology of Aging and Metabolism, University of Minnesota, Minneapolis, MN 55455, USA; 8 Metabolic Section, Department of Medicine, Minneapolis VA Health Care System, Minneapolis, MN 55417, USA; 9 Department of Endocrinology and Diabetes, University of Minnesota, Minneapolis, MN 55455, USA

**Keywords:** fisetin, clinical trials, inflammation, obesity, neurodegeneration, cardiovascular disease, aging, frailty, cancer

## Abstract

Fisetin, a naturally occurring flavonoid, has garnered interest as a potential intervention for age-related diseases due to its anti-inflammatory, antioxidant, and senolytic properties, as demonstrated primarily in preclinical studies. We conducted a structured search and evaluated human translational and clinical studies that utilize fisetin to improve clinical markers associated with age-related low-grade inflammatory diseases. Building on preclinical work and observational studies in humans consuming flavonoid-rich diets or supplements, we evaluated 34 registered clinical trials on ClinicalTrials.gov and the WHO International Clinical Trials Registry Platform (ICTRP) with a focus on aging/frailty, metabolic diseases, cancer, cardiovascular diseases, neuroimmune/neurodegenerative diseases, and other chronic age-related diseases. We provide the status of the 34 registered trials and a synthesis and discussion of the 4 completed trials with available results that included fisetin, identifying gaps and areas for future investigation. Overall, the completed trials indicate that clinical evidence for fisetin remains limited and heterogeneous. While some studies reported favorable changes in metabolic or inflammatory outcomes, others found no clear or significant clinical benefit. Interpretation is further limited by small sample sizes, multimodal interventions, and uncontrolled or exploratory designs, making it difficult to establish fisetin efficacy in humans. Early findings in animal models and in human cell lines suggest that fisetin has the potential to mitigate age-related diseases by targeting chronic inflammation and oxidative stress driven by increased senescent cell burden, but more clinical data are needed.

## 1. Introduction

In this narrative review, we discuss 34 registered clinical trials on the effects of fisetin, primarily in chronic low-grade inflammatory diseases associated with aging. We identified clinical trials in ClinicalTrials.gov and the WHO International Clinical Trials Registry Platform (ICTRP) evaluating the effects of fisetin across different stages of clinical development, including ongoing, completed, suspended, terminated, and withdrawn trials, as well as trials with an unknown recruitment status. We focus our review on synthesizing and discussing registered trials testing fisetin’s effects on aging/frailty, metabolic diseases, cancer, cardiovascular diseases, neuroimmune/neurodegenerative diseases, and other chronic age-related diseases. Most of the described diseases are characterized by imbalances between pro-inflammatory and anti-inflammatory cytokines, and between reactive oxygen species (ROS) production and clearance. As aging is associated with changes in body composition (BC) and the accumulation of senescent cells, this review summarizes what is known from animal and clinical studies on fisetin’s effects on BC and senescent cell clearance. To support the discussion of these trials, we conducted a structured search in other databases, including PubMed/MEDLINE, Scopus, Web of Science (WoS), and Google Scholar. Based on findings from human-derived cell models and animal studies, we discuss the hypothesis that fisetin may reduce the burden of senescent cells and restore oxidative balance, and whether these effects could ultimately contribute to slowing the progression of aging-related diseases in humans.

Overall, 34 registered clinical studies evaluating fisetin were identified, of which 6 were completed and 4 had results available, highlighting the limited availability of human data on fisetin. Nevertheless, these 34 registered trials reflect growing clinical interest in investigating fisetin’s potential geroprotective effects across aging and chronic age-related diseases. The registration of these trials, however, does not itself establish therapeutic efficacy, which remains to be determined as results from ongoing and completed studies become available.

## 2. Fisetin: Interest, Dosing, and Bioavailability as a Nutritional Supplement

Fisetin (3,7,3′,4′-tetrahydroxyflavone, a flavonoid) has emerged as a potential geroprotective molecule capable of mitigating chronic inflammation and oxidative stress. These mechanisms are central to the progression of aging/frailty and chronic diseases in humans, including type 2 diabetes mellitus (T2DM), obesity, cancer, cardiovascular diseases (CVDs), and neurodegenerative diseases [1]. Fisetin is also a senolytic, a class of molecules characterized by their ability to selectively induce cell death in senescent cells, as shown in preclinical models [2].

**Figure 1:**
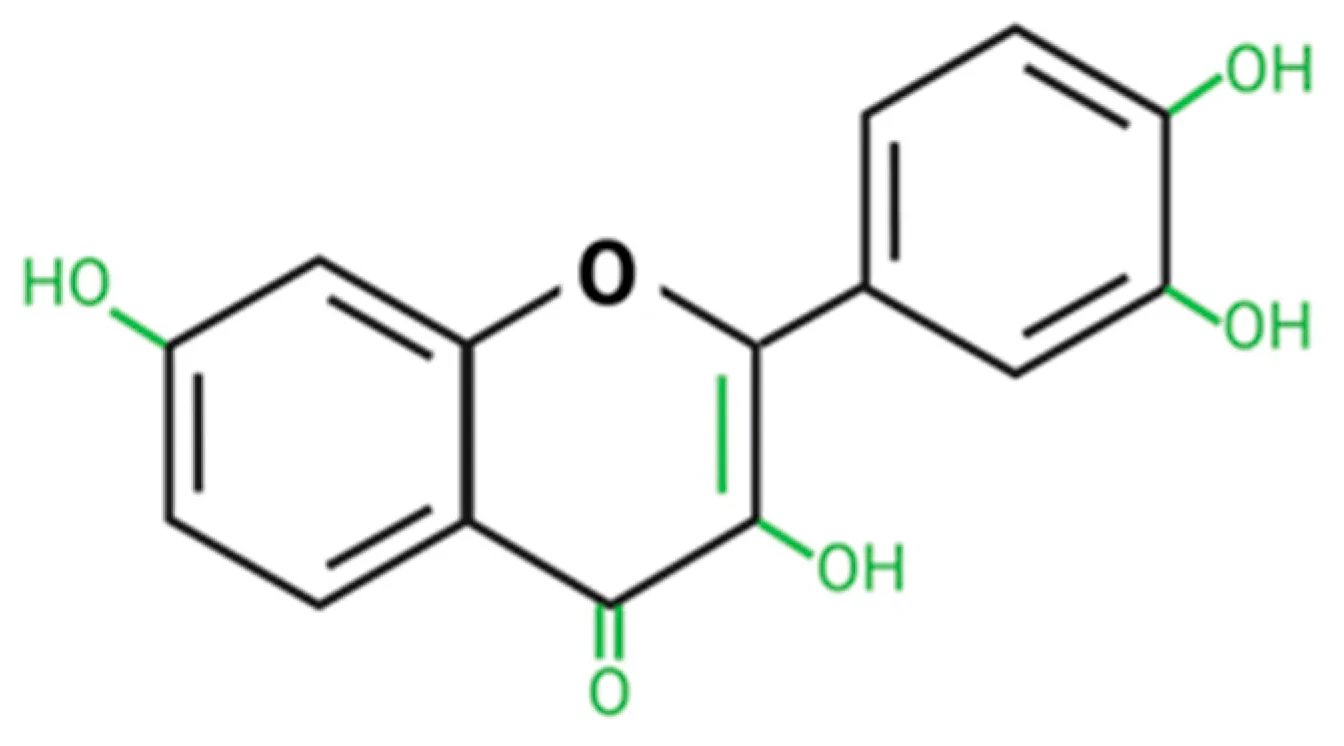
Chemical structure of fisetin. The black backbone corresponds to the basic structure of all flavonoids. The basic flavonoid plus the phenolic hydroxyl groups in the flavonoid structure (highlighted in green) correspond to the chemical structure of fisetin. Adapted from Shin, M.J. et al. [5] and Created in BioRender. Sandoval-Caballero C. (2026) https://BioRender.com/966fdj5.

Fisetin is an organic molecule naturally occurring in vegetables (such as cucumbers and tomatoes) and fruits (such as strawberries and apples) [3]. It belongs to the flavonoid family, characterized by phenolic hydroxyl groups (Figure 1), which contribute to its antioxidant properties [4].

In the United States, fisetin is sold over the counter as a dietary supplement, often taken orally in a wide range of daily doses, from approximately 40–500 mg. Most registered clinical trials have relied on conventional, unformulated fisetin, and the frequency and total single and cumulative doses vary. Only a limited number of studies have incorporated bioavailability-enhancing delivery systems, such as hydrogel formulations, whereas several trial records do not report the formulation administered. Consequently, variation in formulation represents an important source of heterogeneity among clinical studies and should be considered when comparing outcomes and designing future trials. The biological effects of fisetin could persist beyond the day of ingestion. Despite fisetin having a short circulating half-life of approximately 1.14 h [6], its cellular effects persist much longer, lending credence to the idea that some of the effects may be due to a reduction in senescent cellular burden. The plasma half-life of fisetin also varies with the delivery method. In humans, fisetin delivered in a micelle-hydrogel formulation shows much better absorption than unformulated fisetin. After a 1000 mg dose, the hydrogel increased fisetin exposure by ~27-fold (AUC_0–12_ h: 341.4 vs. 12.67 ng·h/mL) and slightly prolonged the half-life (1.51 h vs. 1.14 h [6]) compared to the unformulated version. Overall, these differences in formulation and bioavailability are important considerations when interpreting fisetin clinical trials, as variability in systemic exposure may contribute to differences in efficacy across studies and limit direct comparisons between trials.

### Key Mechanisms of Action—ROS and Inflammation

Reactive oxygen species damage cellular structures, including cell membranes, proteins, and DNA, triggering inflammation and impairing cellular repair mechanisms. During an inflammatory response, various pro-inflammatory cytokines, including tumor necrosis factor-α (TNF-α), interleukin (IL)-1β, IL-4, IL-6, and IL-8, recruit activated immune and inflammatory cells to the lesion site, thereby intensifying and sustaining the inflammatory response [7].

Multi-omic approaches have revealed the molecular mechanisms underlying the antioxidant and antitumorigenic effects of fisetin. For example, fisetin enhances antioxidant enzyme activity to mitigate oxidative stress [8] and reduces several ROS, most notably superoxide (O_2_^•^^−^) and hydroxyl radicals (^•^OH), which contribute to oxidative damage and metabolic dysfunction [9,10,11]. Additionally, fisetin protects cells from hydrogen peroxide (H_2_O_2_)-induced damage, including lipid peroxidation in membranes, DNA damage, and protein carbonylation [12].

In addition, specific intracellular targets for fisetin’s effects are being uncovered. Fisetin has been shown to inhibit the mammalian target of rapamycin (mTOR) signaling pathway, a central regulator of cellular growth, proliferation, survival, and inflammation, with effects reported across diverse cell types, including pancreatic, myeloma, prostate, skin, and liver cancer cells [13,14,15,16,17]. Fisetin’s effects on mTOR signaling are to reduce inflammation [15] through targeting and clearing senescent cells and alleviating chronic inflammation [18]. Additionally, fisetin modulates vascular endothelial growth factor (VEGF), mitogen-activated protein kinase (MAPK), nuclear factor-kappa B (NF-κB), and Nrf2/HO-1 [19]. Taken together, this suggests fisetin may mitigate disease through senolytic and anti-inflammatory activity.

**Figure 2:**
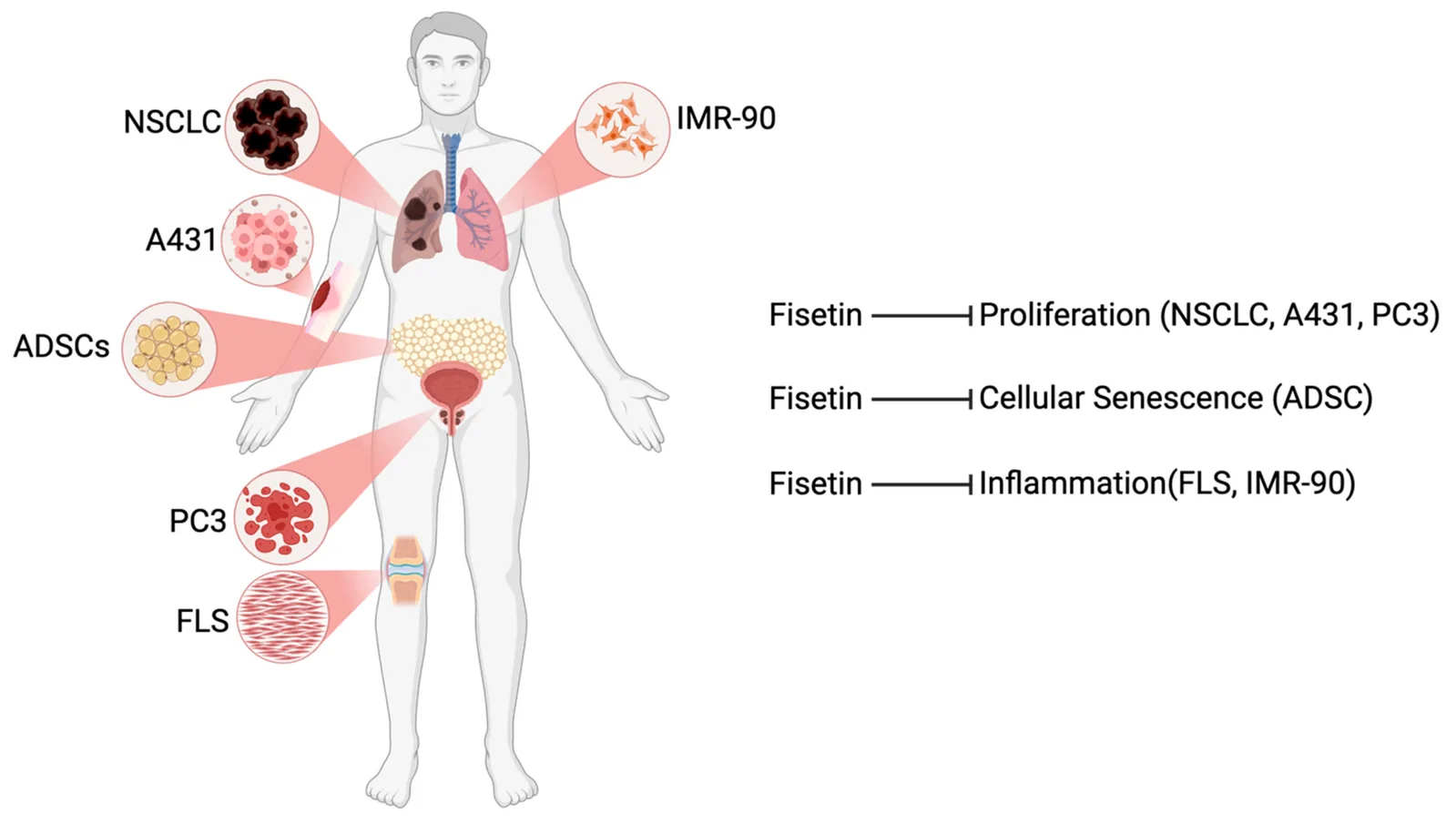
In vitro human cell models used to assess fisetin’s effects on cell proliferation, senescence, and inflammation. The cell models include non-small cell lung cancer (NSCLC) cells, A431 human epidermoid carcinoma cells, PC3 human prostate cancer cells, adipose-derived stem cells (ADSCs), fibroblast-like synoviocytes (FLS), and IMR-90 human lung fibroblasts. Fisetin exhibits anti-neoplastic effects across multiple human cancer cell lines and reduces cellular senescence and inflammation in non-cancer cell types. Together, these findings provide mechanistic and translational rationale for the clinical investigation of fisetin in diseases including type 2 diabetes (T2DM), obesity, cancer, cardiovascular diseases (CVDs), and neurodegenerative disorders; however, these in vitro findings should not be interpreted as evidence of clinical efficacy. Created in BioRender. Sandoval-Caballero C. (2026) https://BioRender.com/vuguktt.

To further confirm the mechanism of action, several different human cell lines have been used in translational studies to define fisetin’s effects (Figure 2). For example, in human fibroblasts (IMR90 cell line) and human adipose tissue explants, fisetin reduces cellular senescence in a dose-dependent manner [18]. It also induces apoptosis in human cervical cancer HeLa cells [20] and in human breast cancer MCF-7 cells [21]. Studies using human-derived in vitro models indicate that fisetin can reduce cellular senescence, suggesting the potential for fisetin to reduce age-related cellular damage in vivo by mechanisms that include increasing the organism’s redox capacity. As individuals age, lipid peroxidation rises due to increased oxidative stress and decreased efficiency of cellular defense mechanisms. In addition, aging can lead to iron accumulation in several tissues, including liver, skeletal muscle, and brain [22]. Early structural studies of fisetin indicate that fisetin modulates lipid peroxidation and increases iron chelation [23]. These preclinical and human-derived in vitro findings provide a mechanistic rationale for investigating whether fisetin can ameliorate aging-related cellular dysfunction in humans, a hypothesis currently being evaluated in clinical studies.

In the following sections, we will examine human translational and emerging clinical trial evidence for fisetin’s geroprotective effects. We will focus our review on studies evaluating fisetin’s effects on aging/frailty, type 2 diabetes/obesity, cancer, cardiovascular disease, and neuroimmune/neurodegenerative diseases. Given the importance of changes in body composition and sarcopenic obesity in the development and exacerbation of chronic age-related diseases, we specifically examined fisetin’s effects on adipose and muscle that could improve age-related changes in body composition.

## 3. A Potential Role in the Prevention of Age-Related Diseases from Dietary Flavonoids—Human Nutritional Observational Studies

Observational studies provide supportive evidence for the potential benefits of flavonoids in general for the chronic age-related diseases that are the focus of this review.

In the Shanghai Women’s Health Study, soy food, but not necessarily the isolated isoflavone component, was inversely associated with postmenopausal bone loss [24], subsequent coronary heart disease (CHD) over 2.5 years of follow-up [25], and premenopausal breast cancer risk [26]. Similarly, a nested case–control study within the Japan Public Health Center-based prospective cohort study showed that greater flavonoid intake was associated with a lower risk of breast cancer [27]. In another Japanese public health center-based prospective study, the incidence of congestive heart failure was lower with some flavonoid-rich fruits [28], and higher flavonoid intake was associated with a reduced risk of stroke in women in this cohort [29].

In studies of European populations, some flavanols were also shown to be protective against incident T2DM [30]. In an Italian study, flavonoid intake was inversely associated with CVDs risk as assessed by the Framingham risk score and with incident events over approximately 10 years of follow-up [31]. The cognitive health benefits of flavonoids were subjectively evaluated in two cohorts of U.S. adults. From 1984 to 2006, 49,493 women from the Nurses’ Health Study (NHS) were followed; 27,842 men from the Health Professionals Follow-Up Study (HPFS) were followed from 1986 to 2002 [32]. This analysis found that flavonoids were associated with better preservation of self-reported cognitive health in these groups. Regarding aggregate study results, one systematic review suggested that a specific class of flavonoids, anthocyanins, may reduce the risk of Parkinson’s, improve motor function [33], and reduce CVDs risk [34]. Finally, a meta-analysis showed that higher flavonoid intake was linked with CVDs prevention [35].

Taken together, these studies suggest that flavonoid intake may exert geroprotective effects and provide a rationale for clinical studies evaluating the efficacy of specific flavonoids, including fisetin, in human diseases. However, because these observational studies evaluated flavonoids broadly rather than fisetin specifically, their findings cannot be directly extrapolated to fisetin.

## 4. Structured Search: Clinical Trials Utilizing Fisetin

This narrative review employs a structured search to identify relevant literature to support the narrative discussion and to search clinical trial registries for the descriptive synthesis. We searched four databases for articles to support the narrative discussion of fisetin’s role in the diseases described above. The databases were PubMed/MEDLINE, Scopus, Web of Science (WoS), and Google Scholar. To retrieve clinical trials evaluating the role of fisetin in the diseases described above, we used ClinicalTrials.gov and the WHO International Clinical Trials Registry Platform (ICTRP; https://trialsearch.who.int/). In all databases, we used the database-specific syntax adapted for each database (see Table 1). The search strategy combined three main concepts: (1) the diseases of interest, (2) fisetin, and (3) human studies, when applicable. Search terms and operators were adapted to the requirements and controlled vocabulary of each database, as described in Table 1.

**Figure 3:**
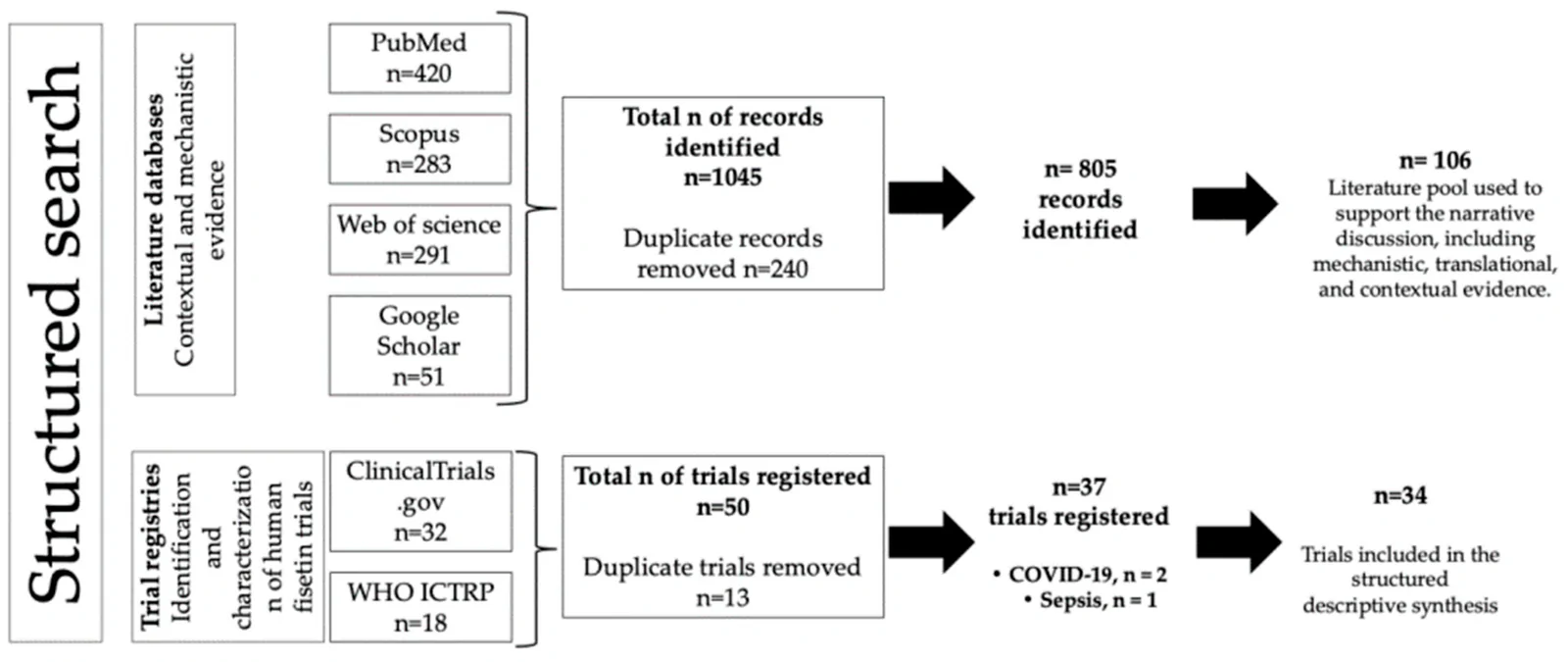
Structured search strategy and use of evidence in the narrative review. A structured search was conducted using four bibliographic databases (PubMed/MEDLINE, Scopus, Web of Science, and Google Scholar) and two clinical trial registries (ClinicalTrials.gov and the WHO International Clinical Trials Registry Platform [ICTRP]). The bibliographic database search identified 1045 records, of which 240 duplicates were removed, yielding 805 unique records. From this literature pool, 106 articles were selected for their relevance to the topics addressed in the narrative review and used to support the mechanistic, translational, and contextual discussion (references in the main text). Separately, the clinical trial registry search identified 50 registered trial records. After removing 13 duplicates, 37 unique registered trials were identified. Three trials were subsequently excluded based on the eligibility criteria (two COVID-19 trials and one sepsis trial), resulting in 34 registered trials included in the structured descriptive synthesis presented in Table 2. The structured search was used to enhance the transparency and reproducibility of the literature and clinical trial identification process and was not intended to constitute a systematic review or meta-analysis.

This article is a narrative review rather than a systematic review or meta-analysis. However, to increase the transparency and reproducibility of the search for literature articles and trials, we conducted a structured search using predefined terms and eligibility criteria (Table 1). The identification, screening, and selection process is summarized in Figure 3.

Among the 34 identified studies evaluating fisetin across different age-related diseases and health outcomes (Table 2), aging/frailty was the most common classification (35.3%), followed by other chronic age-related diseases (23.5%), cancer (14.7%), neuroimmune/neurodegenerative diseases (11.8%), cardiovascular diseases (8.8%), and metabolic diseases (5.9%). The NCT06796374 trial was classified under aging/frailty because it investigates age-related differences in fisetin pharmacokinetics between young and older adults; however, unlike the other studies in this category, its primary focus is pharmacokinetic rather than an aging- or frailty-related clinical outcome.

Phase designation varied across the 34 identified studies. In total, 12 trials (35.3%) were classified as Phase 2, 6 (17.6%) as Phase 1/Phase 2, 2 (5.9%) as Phase 2/Phase 3, 1 (2.9%) as Phase 1, 1 (2.9%) as Early Phase 1, and 1 (2.9%) as Phase 4. In total, 8 studies (23.5%) were classified as N/A (Not applicable), while phase information was unavailable for 3 (8.8%). Overall, Phase 2 was the most common conventional phase designation, indicating that the clinical investigation of fisetin remains relatively early in development, with limited representation of later-phase trials.

This developing evidence base is also reflected in trial status (Table 3). Among the 34 identified studies, 12 (35.3%) were recruiting, and 6 (17.6%) were completed. Of the 6 completed trials, 4 (66.7%) had results available, representing 11.8% (4/34) of all identified studies. In total, 4 studies (11.8%) were active but not recruiting, 4 (11.8%) were withdrawn, and 3 (8.8%) had an unknown status. The remaining studies were enrolled by invitation (2, 5.9%), suspended (1, 2.9%), not yet recruiting (1, 2.9%), or terminated (1, 2.9%).

**Table 2:** General characteristics and classification of the 34 included clinical trials evaluating fisetin in chronic age-related diseases identified through ClinicalTrials.gov and the WHO International Clinical Trials Registry Platform (ICTRP). * Represents clinical trials completed with available results.

Trial ID	Main Disease/Focus	Classification	Trial Name	Source
NCT06630637	Healthy/digital health/intermittent fasting	Aging/frailty	Monitoring Intermittent Fasting for Human Optimization Using Wearable Technology: An N-of-1 Study	ClinicalTrials.gov
NCT07676435	Age-related lung function decline	Aging/frailty	Ongoing Lung Decline with Age Intensified Response	ClinicalTrials.gov and WHO ICTRP
NCT07195318	Healthy aging	Aging/frailty	Fisetin Supplementation for Healthy Aging	ClinicalTrials.gov and WHO ICTRP
NCT07025226	Glioma	Cancer	Medication Combinations of Dasatinib, Quercetin, Fisetin, Temozolomide, LMP744, and Autologous TLPO Vaccine for the Treatment of Previously Treated Glioma with Residual Disease	ClinicalTrials.gov and WHO ICTRP
NCT06990256	Aging/sleep/agingbiomarkers	Aging/frailty	Evaluation of Urolithin A and Fisetin on Improving Sleep and Aging Biomarkers	ClinicalTrials.gov and WHO ICTRP
NCT06819254	Older adult cancer survivors	Cancer	Pilot Study of Fisetin to Improve Fatigue Among Older Adult Cancer Survivors	ClinicalTrials.gov and WHO ICTRP
NCT06399809	Peripheral arterial disease	Cardiovascular diseases	Fisetin to Reduce Senescence and Mobility Impairment in PAD	ClinicalTrials.gov and WHO ICTRP
NCT06113016	Breast cancer survivors	Cancer	Prevention of Frailty with Fisetin and Exercise in Breast Cancer Survivors	ClinicalTrials.gov and WHO ICTRP
NCT05595499	Breast cancer survivors	Cancer	Fisetin to Improve Physical Function in Stage I–III Breast Cancer Survivors	ClinicalTrials.gov and WHO ICTRP
NCT03675724	Frailty in older adults	Aging/frailty	Alleviation by Fisetin of Frailty, Inflammation, and Related Measures in Older Adults	ClinicalTrials.gov and WHO ICTRP
NCT03430037	Frailty in older women	Aging/frailty	Alleviation by Fisetin of Frailty, Inflammation, and Related Measures in Older Women	ClinicalTrials.gov and WHO ICTRP
NCT04770064	Knee osteoarthritis	Other chronic age-related diseases	Targeting Senescence to Reduce Osteoarthritis Pain and cartilagE Breakdown (ROPE)	ClinicalTrials.gov
NCT05505747	Meniscus injury/repair	Other chronic age-related diseases	Enhancing Recovery Through a Combined Mechanobiologic Intervention Following Meniscus Repair	ClinicalTrials.gov
NCT05416515 *	Carpal tunnel syndrome	Other chronic age-related diseases	A Study of Fisetin to Treat Carpal Tunnel Syndrome	ClinicalTrials.gov
NCT06133634	Endothelial dysfunction/arterial stiffness	Cardiovascular diseases	Fisetin to Improve Vascular Function in Older Adults	ClinicalTrials.gov
NCT04733534	Childhood cancer survivors	Cancer	An Open-Label Intervention Trial to Reduce Senescence and Improve Frailty in Adult Survivors of Childhood Cancer	ClinicalTrials.gov
NCT05025956	Femoroacetabular impingement	Other chronic age-related diseases	Senolytic Agent Improve the Benefit of Platelet-Rich Plasma and Losartan	ClinicalTrials.gov
NCT05482672	Knee osteoarthritis/obesity/depression	Other chronic age-related diseases	GetHealthy-OA: A Program to Improve Pain and Function for Patients with Knee Osteoarthritis, Obesity, and Depression	ClinicalTrials.gov
NCT03325322	Diabetes/diabetic nephropathy/CKD	Metabolic diseases	Inflammation and Stem Cells in Diabetic and Chronic Kidney Disease	ClinicalTrials.gov
NCT04313634 *	Age-related skeletal health/cellular senescence	Aging/frailty	Targeting Cellular Senescence with Senolytics to Improve Skeletal Health in Older Humans	ClinicalTrials.gov
NCT04994561	Aging/aging deceleration	Aging/frailty	VIAging Deceleration Trial Using Metformin, Dasatinib, Rapamycin and Nutritional Supplements	ClinicalTrials.gov
NCT04210986 *	Knee osteoarthritis	Other chronic age-related diseases	Senolytic Drugs Attenuate Osteoarthritis-Related Articular Cartilage Degeneration: A Clinical Trial	ClinicalTrials.gov
NCT04815902	Knee osteoarthritis	Other chronic age-related diseases	Use of Senolytic and Anti-Fibrotic Agents to Improve the Beneficial Effect of Bone Marrow Stem Cells for Osteoarthritis	ClinicalTrials.gov
NCT05593588	CVID-associated interstitial lung disease	Other chronic age-related diseases	Senolytics Treatment of Interstitial Lung Disease in Common Variable Immunodeficiency	ClinicalTrials.gov
NCT07678346	Healthy aging/systemic inflammation	Aging/frailty	Evaluation of AppleX™ Apple Extract on Anti-Inflammatory and Healthy Aging Effects	ClinicalTrials.gov
NCT02741804	Mild cognitive impairment	Neuroimmune/neurodegenerative diseases	Lifestyle Intervention Program for Cognitive Impairment	ClinicalTrials.gov
NCT06431932/CTIS2023-506284-34-00	Older adults with multimorbidity/healthy volunteers	Aging/frailty	Pilot Trial of Fisetin in Healthy Volunteers and Older Patients with Multimorbidity	ClinicalTrials.gov and WHO ICTRP
NCT04784234	Primary open-angle glaucoma	Neuroimmune/neurodegenerative diseases	Vision Preservation and Restoration Following a 6-Month Trial of GlaucoCetin	ClinicalTrials.gov
NCT02909686 *	Gulf War Illness/microglial modulation	Neuroimmune/neurodegenerative diseases	Effects of Botanical Microglia Modulators in Gulf War Illness	ClinicalTrials.gov
ISRCTN12085208	Aging markers/vascular risk	Cardiovascular diseases	The effect of a low-calorie Mediterranean diet, intermittent fasting, and natural senolytics on aging markers in participants with low and high vascular risk	WHO ICTRP
IRCT20120129008863N14	Obesity/chronic low-grade inflammation	Metabolic diseases	Comparison of the Efficacy of 12 Weeks of Combined Training with and Without Fisetin Supplementation on the Control of Certain Inflammatory Indices	WHO ICTRP
NCT07279714	Mild Alzheimer’s disease	Neuroimmune/neurodegenerative diseases	Fisetin in Mild Alzheimer’s Disease	WHO ICTRP
ISRCTN10801475	Sarcopenia in later life	Aging/frailty	One study, many treatments: a new way to fight muscle loss in later life	WHO ICTRP
NCT06796374	Fisetin pharmacokinetics in young vs. older adults	Aging/frailty	A Comparison of Fisetin Kinetics in Young and Old Adults	WHO ICTRP

**Table 3:** Intervention characteristics and period, recruitment status, follow-up/outcome, and enrollment of the 34 included clinical trials evaluating fisetin. Note: The intervention period is the interval from initiation to completion of the scheduled intervention and does not necessarily reflect continuous fisetin administration. For intermittent regimens, the Intervention column reports the specific fisetin dosing schedule and number of treatment days. The follow-up/outcome assessment period indicates the reported period over which study outcomes and/or safety assessments were evaluated. BMAC, bone marrow aspirate concentrate. Enrollment (n) indicates actual enrollment when reported for completed, terminated, or withdrawn studies and planned/estimated enrollment for ongoing studies. NR, not reported. * Represents clinical trials completed with available results.

Trial ID	Status	Intervention	Intervention Period	Follow-Up/Outcome Assessment Period	Enrollment (*n*)
NCT06630637	Completed	Multimodal N-of-1 intervention including fisetin (dose and duration not reported), intermittent fasting, dietary interventions, exercise, and other lifestyle interventions | Control: None	Not reported for fisetin; multimodal N-of-1 intervention	Longitudinal assessments throughout the study period	1 actual
NCT07676435	Recruiting	Fisetin ~20 mg/kg/day on days 1–2 and 8–9 (4 doses over 9 days) | Control: Placebo	9 days; fisetin on Days 1–2 and 8–9	NR	40 planned
NCT07195318	Recruiting	Fisetin 100 mg/day | Control: Placebo	7 weeks	7 weeks; outcomes assessed baseline to week 7	120 planned
NCT07025226	Recruiting	Fisetin (dose NR) orally for 2 days per 35-day cycle, alone or with temozolomide or others; follow-up up to 3 years | Control: Other sequential treatment regimens	Variable; repeated 35-day cycles	Up to 3 years; treatment depends on sequential regimen/response	30 planned
NCT06990256	Active not recruiting	Fisetin 500 mg/day or urolithin A 300 mg + fisetin 200 mg/day | Control: Placebo	12 weeks	12 weeks; outcomes assessed through end of treatment	83 planned
NCT06819254	Recruiting	Fisetin 20 mg/kg per dose, twice daily for 2 consecutive days/week | Control: Placebo	6 weeks overall crossover period: 2-week first treatment + 2-week washout + 2-week crossover treatment	12 weeks; post-treatment assessment at Week 12	60 planned
NCT06399809	Recruiting	Fisetin 20 mg/kg/day for 2 consecutive days every 14 days | Control: Placebo	16 weeks/4 months	4 months; primary mobility outcome at 4 months	34 planned
NCT06113016	Recruiting	Fisetin 20 mg/kg/day on days 1–3 every 2 weeks, with/without exercise | Control: Placebo with/without exercise	16 weeks	Up to 3 years; longer-term follow-up follows the 16-week intervention	164 planned
NCT05595499	Recruiting	Fisetin 20 mg/kg/day on days 1–3 every 2 weeks | Control: Placebo	8 weeks	Up to 3 years; longer-term follow-up after the treatment period	88 planned
NCT03675724	Enrolling by invitation	Fisetin 20 mg/kg/day for 2 consecutive days | Control: Placebo	2 days	7 days; primary inflammatory-marker outcome	40 planned
NCT03430037	Enrolling by invitation	Fisetin 20 mg/kg/day for 2 consecutive days/month for 2 consecutive months | Control: Placebo	~2 months	1 month for the registered primary outcome	40 planned
NCT04770064	Withdrawn	Fisetin ~20 mg/kg/day for 2 consecutive days, repeated after 28 days, or 100 mg/day for 90 days | Control: Placebo	90 days (~13 weeks) for low-dose arm; ~1 month for intermittent high-dose arm	3 months	0 actual
NCT05505747	Withdrawn	Fisetin ~20 mg/kg/day for 2 consecutive days, repeated after 28 days | Control: Placebo	~1 month fisetin dosing span, beginning 8 weeks after surgery	1 year for key functional/cartilage outcomes	0 actual
NCT05416515	Completed *	Fisetin ~20 mg/kg/day for 2 consecutive days, repeated after 28 days | Control: none	~1 month dosing schedule	6 months	40 actual
NCT06133634	Active not recruiting	Fisetin 2 mg/kg/day for 3 consecutive days, repeated after 2 weeks (6 dosing days total) | Control: Placebo	~3 weeks from first to final fisetin dose (6 dosing days total)	1 month; primary endothelial-function outcome	70 planned
NCT04733534	Active not recruiting	Fisetin 20 mg/kg/day on days 1, 2, 30, and 31 | Control: Dasatinib 100 mg/day + quercetin 500 mg twice daily on days 1–3 and 30–32	~1 month; fisetin through Day 31	Up to ~5 months	110 planned
NCT05025956	Unknown	Fisetin 20 mg/kg/day for 8 total treatment days | Control: Placebo	~13.4 weeks; final scheduled fisetin dose on Day 94	12 months	68 planned
NCT05482672	Withdrawn	Fisetin 20 mg/kg/day for 8 total treatment days | Control: Placebo	6 weeks; mind–body program, with two fisetin courses during this period	6 months; assessments at baseline, 6 weeks, 3 months, and 6 months	0 actual
NCT03325322	Suspended	Fisetin 20 mg/kg/day for 2 consecutive days | Control: Placebo	2 days	2 weeks	30 planned
NCT04313634	Completed *	Fisetin ~20 mg/kg/day for 3 consecutive days every 4 weeks, repeated 5 times over 20 weeks; or dasatinib + quercetin | Control: Untreated	20 weeks	20 weeks; registry explicitly describes a 20-week trial	74 actual
NCT04994561	Withdrawn	Multimodal regimen including bio-fisetin 44.5 mg, metformin, dasatinib, quercetin, rapamycin, and supplements | Control: None	~12 months after titration/setup; fisetin given 2 days every 3 months ×4	~Day 532 (~76 weeks) for longest safety follow-up	0 actual
NCT04210986	Completed *	Fisetin 20 mg/kg/day for 2 consecutive days, repeated after 28 days | Control: Placebo	~1 month; Days 1–2 and 31–32	12 months for primary safety outcome; monitoring up to 18 months	75 actual
NCT04815902	Unknown	BMAC + fisetin 20 mg/kg for 10 total treatment days, with/without losartan | Control: BMAC + corresponding placebo(s)	~18 weeks from first pre-BMAC fisetin dose through final post-BMAC course	Up to 18 months for patient-reported outcomes	100 planned
NCT05593588	Active not recruiting	Fisetin 20 mg/kg/day on days 0, 1, 28, and 29 | Control: Placebo	~1 month; Days 0–1 and 28–29	6 months	20 planned
NCT07678346	Not yet recruiting	AppleX™ apple extract containing fisetin | Control: Placebo	24 weeks	24 weeks	70 planned
NCT02741804	Unknown	BBH-1001 supplement containing fisetin 16.65 mg/soft gel (66.6 mg/day = 4 soft gel/day) + other micronutrients + lifestyle intervention | Control: Placebo + lifestyle intervention	18 months	18 months for main outcomes; extended dementia follow-up planned for 7–10 years	150 planned
NCT06431932/CTIS2023-506284-34-00	Recruiting	Fisetin 20 mg/kg/day for 2 consecutive days | Control: Placebo (older-patient randomized cohort); none (healthy-volunteer cohort)	2 days	24 h for the primary pharmacokinetic outcome; additional safety/biomarker assessments are also planned	60 planned
NCT04784234	Terminated	GlaucoCetin containing fisetin (dose not reported), 6 capsules/day for 6 months | Control: Placebo	6 months	6 months	12 actual
NCT02909686	Completed *	Fisetin 200–800 mg/day | Control: Placebo	~17 weeks per botanical sequence	~17 weeks per sequence; potentially longer for participants completing additional botanical sequences	36 actual
ISRCTN12085208	Recruiting	Fisetin 20 mg/kg/day for 2 consecutive days/month + low-calorie Mediterranean diet + intermittent fasting, for ≥2 years | Control: Mediterranean diet + placebo	≥2 years (≥104 weeks)	≥2 years; regular assessments throughout	4000 planned
IRCT20120129008863N14	Recruiting	Fisetin 200 mg/day with/without combined exercise training | Control: Placebo with/without combined exercise training	12 weeks	12 weeks; pre/post intervention assessments	44 planned
NCT07279714	Completed	Fisetin 20 mg/kg/day | Control: None	NR for exact dosing duration	30 days for the registered primary safety/tolerability outcome	5 actual
ISRCTN10801475	Recruiting	Multiple platform treatments, including fisetin | Control: Usual care	12 weeks; fisetin for 3 consecutive days every 2 weeks	12 weeks	36 planned
NCT06796374	Recruiting	Fisetin 500 mg single oral dose (fed) | Control: Fisetin 500 mg single oral dose (fasted)	Single oral dose per fed/fasted study condition	10 h for the primary pharmacokinetic assessment	80 planned

Regarding fisetin formulation (Table 3), most studies administered fisetin as the principal intervention, generally reporting the dose in mg/day or mg/kg/day without specifying the dosage form, although fisetin was likely administered orally as a pill, capsule, or tablet in many of these studies. Several studies evaluated fisetin as part of multi-component or proprietary interventions, including Bio-fisetin within a multimodal regimen (NCT04994561), the BBH-1001 multi-ingredient supplement (NCT02741804), GlaucoCetin (NCT04784234), AppleX™ apple extract (NCT07678346), and a lifestyle intervention including fisetin (NCT06630637). NCT06990256 also included an intervention arm combining fisetin with urolithin A. Among these six multi-component or proprietary interventions, the amount of fisetin was reported in only three, ranging from 44.5 to 200 mg: NCT04994561 included 44.5 mg of Bio-fisetin, NCT02741804 provided 66.6 mg/day of fisetin through BBH-1001, and NCT06990256 combined 200 mg/day of fisetin with 300 mg/day of urolithin A. In contrast, the fisetin content of GlaucoCetin, AppleX™, and the multimodal intervention in NCT06630637 was not reported.

Taken together, these findings indicate that the clinical evidence for fisetin remains preliminary and highly heterogeneous. Although the growing number of trials across diverse diseases demonstrates broad interest in its potential clinical applications, the predominance of ongoing studies, limited number of completed and later-phase trials, and substantial variability in dosing regimens, formulations, and co-interventions currently limit direct comparisons across studies and preclude firm conclusions regarding clinical efficacy. Importantly, incomplete reporting of fisetin formulation represents an additional translational challenge, particularly given the potential influence of formulation on bioavailability and systemic exposure. Thus, larger, well-controlled clinical trials using clearly defined and standardized fisetin formulations and dosing regimens are needed to determine its efficacy, safety, and clinical relevance, particularly in the context of aging and age-related diseases.

Given the still emerging clinical evidence, a broader evaluation of the literature is needed to contextualize the potential effects of fisetin across these diseases. The following sections therefore examine the available evidence for fisetin in aging/frailty, metabolic diseases, cancer, cardiovascular diseases, and neuroimmune/neurodegenerative diseases, integrating findings from observational studies, preclinical models, and human cell-based studies. These complementary levels of evidence provide important mechanistic and biological context for the clinical findings while recognizing that effects observed in experimental models do not necessarily translate into clinical efficacy in humans. Within each disease, this evidence is discussed alongside the available clinical trials to highlight both the current evidence supporting fisetin and the remaining gaps in its clinical translation.

## 5. Aging/Frailty

Aging is a natural process associated with increased cellular senescence, chronic inflammation, and heightened oxidative stress, collectively termed “inflammaging” [36]. At 35.3%, more than one-third of fisetin studies indexed/posted on ClinicalTrials.gov or the WHO International Clinical Trials Registry Platform (ICTRP; Table 2) as of August 2026 were classified as aging-/frailty-related. These studies investigate diverse aspects of aging and frailty, including bone resorption and formation (NCT04313634), physical function and frailty (NCT03675724 and NCT03430037) among other aging-related outcomes. These biological processes are major contributors to age-related declines in muscle mass and function, bone loss, and frailty.

Given that greater lean mass, which partly reflects skeletal muscle mass, has been associated with longevity [37], and sarcopenia is associated with falls, fractures, metabolic disorders, cognitive impairment, and increased mortality in the general population [38,39,40], the maintenance of muscle mass and function has been identified as a key target in preventing frailty.

Fisetin’s mechanism of action influences several cellular processes that could mitigate frailty by protecting muscle function. Healthy skeletal muscle mass and function protect bone health, which supports movement and posture. Muscle is a key site for glucose uptake and fat oxidation. Healthy muscle maintains protein synthesis and repair processes, neutralizing ROS by antioxidant systems to preserve function. High levels of ROS can negatively affect muscle metabolism and myogenesis [41] through NF-κB, and p38 MAPK pathways [42], oxidative stress, muscle damage [41], inflammation [43], and the regulation of muscle protein synthesis [44]. Myocytes consume large amounts of oxygen through aerobic respiration, especially during exercise, a period associated with high ROS generation [45], which may be modulated by fisetin supplementation.

In addition, fisetin and other flavonoids’ suppressive effects on TNF-α and IL-6, two key inflammatory cytokines, could directly improve muscle protein metabolism [46,47]. Elevated levels of pro-inflammatory cytokines TNF-α and IL-6 activate NF-κB, which in turn suppresses protein synthesis and increases proteolysis [48]. This leads to muscle atrophy [43,44] and to the chronic, progressive form of muscle atrophy, sarcopenia [49]. By suppressing macrophage TNF-α and IL-6 expression, fisetin may reduce the inflammatory burden on muscle tissue, thereby improving protein homeostasis and promoting the clearance of senescent immune cells. This, in turn, facilitates muscle regeneration and improves dystrophic muscle phenotypes [50,51]. These latter findings were obtained from preclinical models; however, one cannot assume the same mechanisms operate in humans.

One aspect of the aging process in muscle is that muscle stem cells progressively lose their ability to proliferate or differentiate [52]. While evidence in humans is limited, fisetin has been shown to improve physical function and reduce cellular senescence in the skeletal muscle of aged mice, and to protect C2C12 mouse myoblasts from oxidative stress-induced injury [53]. Also, its metabolite Geraldol (3,4′,7-trihydroxy-3′-methoxyflavone) [54] could inhibit human lipoxygenase, hydroxysteroid dehydrogenase, and Caspase-1 enzymes, suggesting potential anti-inflammatory and cytoprotective activities in addition to its reported pro-proliferative effect in human satellite cells [55]. Overall, although there is currently no direct evidence that fisetin itself promotes the proliferation of human skeletal muscle stem cells (satellite cells), the observed effects of its metabolite Geraldol provide preliminary evidence that fisetin-derived metabolites may influence satellite-cell biology in humans.

The available evidence from human stem cells is limited to non-muscle cell types, such as adipose-derived stem cells. In animal models, fisetin mitigates ROS-mediated damage in myoblasts subjected to oxidative damage [53] and the adverse changes in frailty and grip strength associated with aging [56,57]. Although more work is needed in human myocyte cell lines to substantiate fisetin’s effects on muscle health, recent murine data demonstrate that fisetin enhances skeletal muscle function [56] and clears senescent cells from muscle tissue, thereby potentially promoting myogenesis [18,51]. Overall, this proof-of-concept evidence in animal models provides a rationale for investigating whether fisetin can reduce senescent-cell burden in skeletal muscle and preserve muscle function in humans [56,57].

### Clinical Trials: Aging/Frailty and Fisetin

Among the 4 completed clinical trials with available results, 1 evaluating aging- or frailty-related outcomes (NCT04313634), evaluated skeletal health and cellular senescence in older adults (Table 2 and Table 3). Although human evidence remains limited, emerging evidence suggests that fisetin may help mitigate age-related inflammation and functional decline, which contribute to frailty. In a separate study, individuals with mild to moderate osteoarthritis exhibited elevated levels of senescent peripheral blood mononuclear cells (PBMCs) and T cells, as identified by C12FDG (5-Dodecanoylaminofluorescein di-β-d-galactopyranoside) fluorescence. Fisetin reduced both C12FDG+ PBMCs and circulating senescence-associated biomarkers [58]. These findings are consistent with in vitro evidence showing that fisetin inhibits the IL-1β-induced inflammatory response in human osteoarthritis chondrocytes, potentially alleviating the progression of osteoarthritis [59]. Together, these findings support the hypothesis that fisetin may influence age-related diseases by targeting senescent cells and thereby potentially reducing circulating pro-inflammatory markers. These findings provide a rationale for ongoing trials evaluating the clinical effects of fisetin across a range of aging- and frailty-related outcomes. As summarized in Table 3, current and completed trials are evaluating or have evaluated outcomes related to frailty and physical function (NCT03675724 and NCT03430037), skeletal health and cellular senescence (NCT04313634), healthy aging and aging-related biomarkers (NCT07195318 and NCT06990256), and sarcopenia in later life (ISRCTN10801475). Other trials have extended this investigation to chronic age-related diseases, including osteoarthritis (NCT04770064 and NCT04210986).

The “Targeting Cellular Senescence with Senolytics to Improve Skeletal Health in Older Humans” trial (NCT04313634) is the only trial completed with available results that allows the effects of fisetin to be evaluated independently. This 20-week study compared dasatinib plus quercetin, fisetin, and an untreated control to investigate whether senolytic therapies could reduce cellular senescence and improve skeletal health in older adults. Outcomes included bone turnover markers (CTX and P1NP), bone mineral density measured by DXA, and senescence biomarkers. The results regarding the effect of fisetin were inconclusive, as only 11 participants who received fisetin were included in the analysis, and a larger sample size will be needed. Results from the other completed aging-/frailty-related trial (NCT06630637) are not available in ClinicalTrials.gov but were recently published in August 2026 [60]. This prospective, open-label N-of-1 study evaluated a multimodal health intervention in a single healthy adult. The study reported changes in cardiometabolic biomarkers, metabolic flexibility, and other measures of biological resilience. Fisetin was included as part of the broader multimodal intervention, but its specific effects were not evaluated, and cellular senescence was not directly assessed. Therefore, the reported outcomes cannot be attributed specifically to fisetin.

Despite the limited evidence from completed studies, several ongoing trials may help clarify the potential role of fisetin in aging and frailty. The NCT03675724 and NCT03430037 trials are particularly relevant because they directly evaluate frailty- and inflammation-related outcomes in older adults and may help determine whether the proposed senolytic effects of fisetin translate into measurable changes in physical function and systemic inflammation. Other trials broaden this investigation to specific manifestations of aging, including age-related lung function decline (NCT07676435), sarcopenia in later life (ISRCTN10801475), and healthy aging and aging-related biomarkers (NCT07195318 and NCT06990256). In addition, the NCT06431932 and NCT06796374 trials may provide information regarding the pharmacokinetics, safety, and biological effects of fisetin in older populations. Collectively, these studies may help determine whether fisetin-induced changes in cellular senescence and inflammatory biomarkers are accompanied by clinically meaningful improvements in physical and physiological function. Their results will therefore be important for establishing whether the biological effects observed in preclinical models translate into geroprotective effects in humans.

## 6. Metabolic Diseases—Type 2 Diabetes Mellitus (T2DM) and Obesity

The accumulation of ROS is a hallmark of cellular senescence in human adipose-derived stem cells (ADSCs), a population of mesenchymal stem cells (MSCs). Increased oxidative stress may impair ADSC regenerative potential and drive the secretion of senescence-associated inflammatory factors, thereby exacerbating oxidative stress and tissue dysfunction [61]. In cultured human ADSCs, fisetin treatment reduces lipid peroxidation, protein oxidation, and mitochondrial dysfunction, all of which are critical for the health of adipose tissue. These findings suggest that fisetin may help attenuate oxidative stress and cellular senescence in ADSCs; however, whether these effects translate into improved adipose tissue function or protection against metabolic diseases in humans remains to be established. Below, we discuss the available evidence on the effects of fisetin on adipose tissue.

Adipogenesis, the formation of new adipocytes, when dysregulated, contributes to obesity and T2DM when excessive [62]. Adipose tissue regulates energy storage and metabolism through triglyceride turnover and the secretion of hormones such as leptin and adiponectin [63]. Under healthy conditions, oxidative stress and inflammation are minimal; however, increased oxidative stress promotes adipocyte differentiation and lipid accumulation, leading to fat mass expansion and dysfunction [64]. Inflammation further drives this process, as macrophage infiltration elevates TNF-α and IL-6 levels, thereby disrupting insulin signaling and promoting adipocyte hypertrophy [65]. Collectively, these mechanisms link oxidative and inflammatory stress to adipose dysfunction, suggesting that reducing inflammation with fisetin could potentially offer a new therapeutic approach to obesity.

Fisetin’s effects on adipogenesis may, in part, be mediated by its actions on mTOR. The mechanistic target of rapamycin, composed of two distinct multiprotein complexes—mTOR complex 1 (mTORC1) and mTOR complex 2 (mTORC2)—is critical in adipose tissue regulation, influencing adipogenesis, lipid metabolism, thermogenesis, and adipokine homeostasis [66]. Activation of mTOR signaling increases fat mass (enhancing adipogenesis and lipogenesis, and blocking lipolysis [67]), which in turn has been associated with increased adiposity and insulin resistance. Inhibition of mTORC1 also inhibits adipocyte differentiation and adipogenesis in human preadipocytes, while increasing mTORC1 signaling promotes these processes. This suggests that modulation of mTORC1 may influence adipose tissue expansion and metabolic function [68,69]. In humans, the mTORC1 signaling effector ribosomal protein S6 kinase (S6K) is upregulated in the visceral fat of subjects with obesity and is associated with insulin resistance and inflammation [70]. Preclinical data have shown that fisetin attenuated diet-induced weight gain in mice [71] and senescence in ADSCs [61]. Although fisetin has been shown to inhibit mTORC1 signaling in murine 3T3-L1 preadipocytes [71], its effects on mTOR signaling have not been directly examined in human adipocytes. In 3T3-L1 preadipocytes, fisetin suppressed the expression of proteins involved in adipocyte differentiation, including peroxisome proliferator-activated receptor γ (PPARγ). Importantly, fisetin did not induce significant cytotoxicity in 3T3-L1 preadipocytes at the concentration used to examine these effects, suggesting that the observed inhibition of adipogenesis was not simply attributable to reduced cell viability [71].

Evidence from human-derived cells, however, is less consistent. In a study utilizing human adipose-derived stem cells (ADSCs), fisetin treatment largely preserved adipogenic differentiation capacity, with no significant changes in PPARγ, C/EBPα, FABP4, or lipid accumulation in low-passage cells [61]. Although PPARγ expression was reduced in high-passage ADSCs, other adipogenic markers and lipid accumulation remained unchanged. However, in contrast, a 2024 study using human mesenchymal stromal cells reported that fisetin promoted adipogenic differentiation [72]. Specifically, fisetin increased lipid accumulation and the expression of PPARγ, as well as of genes involved in triglyceride synthesis, lipid-droplet budding, and lipid-droplet fusion. Although these studies differ not only in species but also in cell type, differentiation stage, and experimental conditions, their diverging findings suggest that the effects of fisetin on adipogenesis may be highly context-dependent. These diverging findings further underscore the need for caution when extrapolating the effects of fisetin observed in murine models to humans and reinforce the importance of validating preclinical findings in physiologically relevant human models.

One important aspect of fisetin’s actions may be its potential to modulate the local tissue environment by reducing senescence-associated paracrine signaling. Cellular senescence can disrupt communication among neighboring cell populations through the senescence-associated secretory phenotype (SASP). Consistent with this mechanism, animal studies have demonstrated that Fisetin reduces senescent-cell burden and SASP factors in some tissues, thereby promoting geroprotection. Fisetin treatment in aged mice was shown to attenuate aberrant signaling from senescent endothelial cells to other vascular cell populations, including CXCL12-mediated intercellular communication, and to reduce the detrimental effects of the circulating SASP milieu on endothelial function [18]. These findings suggest that fisetin-mediated senescent-cell clearance may improve tissue function, at least in part, by reducing maladaptive paracrine signaling and restoring a more favorable local cellular environment. The presence of dysfunctional senescent adipose tissue could negatively impact muscle function and contribute to muscle atrophy [44]. As adipocytes become hypertrophic and adipose tissue becomes dysfunctional, they exhibit increased secretion of pro-inflammatory factors, including TNF-α and IL-6, which can contribute to chronic low-grade inflammation [73]. This inflammatory environment may disrupt normal muscle metabolism by increasing protein degradation and reducing protein synthesis, thereby promoting muscle degradation [74,75]. Over time, these factors can lead to sarcopenic obesity, the muscle loss and decreased function sometimes observed in obesity [76]. Thus, inflammation, oxidative stress, and metabolic dysfunction may contribute to maladaptive crosstalk between adipose tissue and muscle, creating a vicious cycle that exacerbates muscle atrophy and further deteriorates muscle function.

### Clinical Trials: T2DM/Obesity and Fisetin

Despite substantial preclinical interest in the metabolic effects of fisetin, only 5.9% (2/34) of the registered clinical trials identified were primarily classified as investigating metabolic diseases, including obesity and T2DM (Table 2). Although relatively few fisetin trials primarily target metabolic diseases, several trials across disease categories include metabolic outcomes relevant to obesity and T2DM, offering an opportunity to examine fisetin’s potential effects on senescence, inflammation, and metabolic dysfunction. In particular, the “Alleviation by Fisetin of Frailty, Inflammation, and Related Measures in Older Women” trial (NCT03430037) and the related “Alleviation by Fisetin of Frailty, Inflammation, and Related Measures in Older Adults” trial (NCT03675724), both classified under aging/frailty in our analysis, evaluate markers of insulin resistance and inflammation in addition to their primary aging- and frailty-related outcomes. Thus, although these studies were not specifically designed as obesity or T2DM trials, they may provide complementary information regarding the potential metabolic effects of fisetin in older adults.

Of the two trials classified as investigating metabolic diseases, IRCT20120129008863N14 was listed as recruiting (50%, 1/2), whereas NCT03325322 was suspended (50%, 1/2) due to a lack of funding and therefore has not generated completed clinical evidence. Although IRCT20120129008863N14 was listed as recruiting in the WHO ICTRP at the time of our search, results from this trial have been published, indicating completion of the reported 12-week intervention. This trial evaluated fisetin at 200 mg/day, alone or in combination with exercise training, for 12 weeks in men with obesity and reported improvements in body weight and inflammatory and metabolic outcomes, with several outcomes showing greater improvements when fisetin was combined with exercise [77]. Thus, although one of the two metabolic trials has generated preliminary human efficacy data, the very small number of trials and the suspended status of the only trial specifically targeting individuals with diabetes highlight the limited clinical evidence currently available for fisetin in obesity and T2DM. Moreover, because fisetin was evaluated both alone and in combination with exercise training, the extent to which the reported benefits can be attributed specifically to fisetin remains uncertain.

Complementary evidence from our literature search suggests that fisetin may affect body composition and metabolic outcomes in individuals with T2DM or obesity. In a separate study of individuals with T2DM, fisetin at 100 mg/day for 8 weeks, in addition to metformin therapy, significantly reduced body weight, BMI, and waist circumference compared with metformin alone and improved insulin resistance and adipokine profiles [78]. Together, these studies provide preliminary evidence that fisetin may influence body composition, inflammation, and metabolic health in humans. However, the evidence remains limited by relatively small sample sizes, short intervention periods, and restricted study populations. Moreover, fisetin was administered alongside exercise training or metformin, complicating the attribution of the observed effects specifically to fisetin. Larger, longer-term randomized, placebo-controlled trials are therefore needed to establish the efficacy and safety of fisetin in metabolic diseases.

## 7. Cancer

Preclinical studies in human cancer cell lines have demonstrated the anticancer effects of fisetin [8,13,15,17,21,79,80,81,82,83], providing a biological rationale for further investigation of its potential in clinical trials. Consistent with this preclinical interest, several clinical trials have subsequently evaluated or are currently evaluating fisetin in cancer-related settings. Fisetin has shown anti-neoplastic effects in human cancer cell lines, including lung cancer [80], epidermoid carcinoma [84], and prostate cancer cells [82]. Chronic inflammation and oxidative stress are significant contributors to cancer development and progression. TNF-α and IL-6 promote tumor cell growth via activation of STAT3 and NF-kB in experimental models, and in patients, elevated circulating IL-6 and TNF-α levels are associated with more advanced disease and poorer prognosis [85,86]. Additionally, ROS can cause DNA damage, leading to mutations that initiate cancer development [87]. Fisetin’s senolytic properties hold promise for potential cancer treatment regimens. Several studies have demonstrated that fisetin inhibits cellular proliferation and/or induces apoptosis in hepatic, gastric, breast, colorectal, and pancreatic cancer cells [21,83,88]. The ability of fisetin to influence several cancer-related pathways may also make it of interest as an adjunct to established anticancer therapies rather than solely as an independent intervention.

Consistent with this possibility, fisetin has been investigated in combination with other anticancer drugs in preclinical models. In human cervical cancer cells, fisetin combined with sorafenib produced greater pro-apoptotic effects than either agent alone, with complementary in vivo pro-apoptotic effects providing additional support for the combination [81]. Such findings raise the possibility that fisetin could modify tumor-cell responses to conventional therapies. However, synergistic effects observed in cellular and preclinical models cannot be assumed to translate to improved therapeutic efficacy or safety in patients.

Fisetin’s senolytic activity provides an additional rationale for its investigation in cancer, as cellular senescence plays a role in cancer: although senescence can initially suppress tumorigenesis by limiting the proliferation of damaged cells, persistent senescent cells can secrete SASP factors that modify the tumor microenvironment and may contribute to chronic inflammation and tumor progression. Cancer therapies themselves can also induce cellular senescence in both tumor and non-tumor cells. Consequently, the ability of senolytic compounds such as fisetin to selectively reduce specific senescent cell populations has generated interest in their potential use in cancer treatments. Nevertheless, because senescence can have both tumor-suppressive and tumor-promoting functions, the consequences of senescent-cell clearance in cancer are likely to depend on tumor type, disease stage, treatment context, and the specific cell populations affected.

Collectively, these preclinical findings provide a biological rationale for investigating fisetin in cancer but should not be interpreted as evidence of clinical anticancer efficacy. Consistent with this preclinical interest, fisetin is being evaluated in clinical studies to assess its potential in some cancers described in the following section.

### Clinical Trials: Cancer and Fisetin

Among the registered clinical trials identified, 14.7% (5/34) were classified as investigating cancer (Table 2). Most of these studies evaluate fisetin in the context of cancer survivorship, with a particular focus on physical function, frailty, fatigue, inflammation, and cellular senescence following cancer treatment (NCT06113016, NCT05595499, NCT04733534, and NCT06819254), whereas one trial evaluates fisetin as part of a multimodal therapeutic strategy for patients with previously treated glioma and residual disease (NCT07025226).

Four of the five cancer-related trials are currently recruiting. The “Prevention of Frailty with Fisetin and Exercise (PROFFi) in Breast Cancer Survivors” trial (NCT06113016) evaluates fisetin, alone or in combination with exercise, in breast cancer survivors, with outcomes related to frailty, physical function, inflammation, and cellular senescence. The “Fisetin to Improve Physical Function in Stage I–III Breast Cancer Survivors” trial (NCT05595499) similarly evaluates fisetin in breast cancer survivors, focusing on physical function and other treatment-related outcomes. The “Pilot Study of Fisetin to Improve Fatigue Among Older Adult Cancer Survivors” (NCT06819254) evaluates whether fisetin can reduce fatigue in older cancer survivors. In a different clinical context, the early-phase “Medication Combinations of Dasatinib, Quercetin, Fisetin, Temozolomide, LMP744, and Autologous TLPO Vaccine for the Treatment of Previously Treated Glioma with Residual Disease” trial (NCT07025226) evaluates fisetin as part of sequential or combination treatment regimens in patients with previously treated glioma and residual disease. Because fisetin is administered within a multimodal therapeutic strategy in this study, any observed effects cannot necessarily be attributed to fisetin alone.

The remaining cancer-related trial, NCT04733534, is active but not currently recruiting. The “Open-Label Intervention Trial to Reduce Senescence and Improve Frailty in Adult Survivors of Childhood Cancer” evaluates senolytic interventions, including fisetin, in adult survivors of childhood cancer, with outcomes related to cellular senescence, frailty, inflammation, and physical function.

Overall, 80% (4/5) of the cancer-related trials are recruiting, and 20% (1/5) are active but not recruiting. None of the five cancer-related trials classified in our analysis are currently listed as completed; therefore, no completed clinical evidence from these registered trials is yet available to establish the efficacy of fisetin in cancer treatment or survivorship.

## 8. Cardiovascular Diseases (CVDs)

Cardiovascular diseases (CVDs), including coronary and peripheral artery disease (PAD), heart failure, and stroke, are major contributors to morbidity and mortality worldwide [89]. Chronic inflammation and oxidative stress are key drivers in the pathogenesis of CVDs. Elevated levels of pro-inflammatory markers, such as C-reactive protein (CRP) and cytokines like IL-6 and TNF-α, have been linked to increased risk and progression of CVDs [89]. Fisetin may influence cardiovascular health and prevent the progression of CVDs by reducing the senescent cellular burden, lowering inflammatory cytokines, maintaining endothelial function, reducing plaque formation, and minimizing vascular damage. This rationale is supported by in vitro studies demonstrating that fisetin acts as a senolytic in human cells. For example, fisetin selectively induced apoptosis/reduced viability in senescent human umbilical vein endothelial cells (HUVECs) compared with proliferating HUVECs, directly supporting the designation of fisetin as a senolytic in human endothelial cells [90]. Beyond endothelial cells, vascular smooth muscle cells (VSMCs) represent another important cellular target because they contribute to vascular tone and structural integrity, while their senescence is associated with inflammation and pathological vascular remodeling [91]. More recent preclinical evidence further demonstrates that intermittent fisetin treatment reduces vascular and endothelial-cell senescence and SASP signaling in aged mice, with complementary experiments in human aortic endothelial cells implicating CXCL12-mediated senescence, mitochondrial oxidative stress, and impaired NO bioavailability in age-related endothelial dysfunction [92]. These vascular cell types are particularly relevant to CVDs, as endothelial dysfunction, vascular inflammation, and accumulation of senescent cells contribute to CVDs. While human cardiomyocytes and cardiac fibroblasts have not yet been tested with fisetin, an in silico drug-repositioning approach identified fisetin as a candidate associated with transcriptional signatures of cardiac regeneration [93]. Subsequent studies in rat cardiomyocytes showed that fisetin reduced ROS production and apoptosis and improved cell survival following hypoxia/reoxygenation. Whether these cardioprotective effects extend to human cardiomyocytes remains unknown [93]. It should be noted that in vitro and in silico studies do not capture the full complexity of cardiac tissue (e.g., the microenvironment, cell–cell interactions, and crosstalk), which can influence the effects of fisetin. In addition, dosing, pharmacokinetics, and bioavailability in humans may differ significantly from experimental conditions, underscoring the need for further research.

Beyond its effects on cellular senescence, preclinical studies suggest that fisetin may influence additional mechanisms involved in cardiovascular injury. In an ex vivo isolated-heart model of myocardial ischemia/reperfusion injury, fisetin attenuated myocardial injury and preserved mitochondrial structure and bioenergetic function [94], suggesting a potential role in maintaining cardiac mitochondrial homeostasis. At the vascular level, fisetin reduced atherosclerotic lesion formation in ApoE^−/−^ mice (a model of atherosclerosis characterized by impaired lipoprotein clearance, hypercholesterolemia, and spontaneous development of atherosclerotic lesions), accompanied by reductions in circulating total cholesterol, triglycerides, and LDL cholesterol, and increased cholesterol excretion [95].

Collectively, these findings suggest that the potential cardiovascular effects of fisetin may extend beyond senescent-cell clearance and involve interconnected mechanisms, including modulation of oxidative stress and inflammation, preservation of endothelial and mitochondrial function, and regulation of lipid metabolism and atherosclerotic pathology. Together, these preclinical findings provide a biological rationale for investigating whether these effects translate into cardiovascular benefits in humans, as currently being explored in clinical trials.

### Clinical Trials: CVDs and Fisetin

Among the registered clinical trials identified, 8.8% (3/34) were classified as investigating CVDs (Table 2), including peripheral artery disease (NCT06399809), endothelial dysfunction and arterial stiffness (NCT06133634), and vascular risk and aging-related markers (ISRCTN12085208). Together, these trials evaluate different aspects of cardiovascular health, ranging from vascular function and mobility impairment in individuals with established vascular disease to vascular aging and cardiovascular risk.

Two of the three cardiovascular trials are currently recruiting (66.7%, 2/3). The “Fisetin to Reduce Senescence and Mobility Impairment in PAD” trial (NCT06399809) investigates whether intermittent fisetin treatment can reduce cellular senescence and improve mobility in individuals with peripheral artery disease. ISRCTN12085208 evaluates a longer-term intervention combining fisetin with a low-calorie Mediterranean diet and intermittent fasting in individuals with different levels of vascular risk. Because fisetin is administered as part of a multimodal intervention in this study, any observed effects cannot necessarily be attributed to fisetin alone.

The remaining trial, “Fisetin to Improve Vascular Function in Older Adults” (NCT06133634), is active but not recruiting. This placebo-controlled study evaluates whether fisetin can improve endothelial function in older adults, with a particular focus on vascular function and arterial stiffness. Thus, this trial represents a clinical investigation of the preclinical evidence linking cellular senescence to age-related endothelial dysfunction.

None of the three cardiovascular trials included in our analysis have been completed; therefore, no completed clinical evidence from these registered trials is yet available to establish whether fisetin improves cardiovascular outcomes in humans. Although NCT06133634 is not yet listed as completed, preliminary findings from this trial have been reported in the form of a conference abstract [96]. In 41 older adults, fisetin treatment (2 mg/kg/day) improved brachial artery flow-mediated dilation by approximately 41% compared with baseline, whereas no improvement was observed with placebo. These changes were accompanied by reductions in markers of cellular senescence and mitochondrial oxidative stress, providing preliminary human evidence that fisetin may improve vascular endothelial function. However, these findings have been reported only as a conference abstract to date and should therefore be interpreted with caution until complete peer-reviewed results are available.

## 9. Neuroimmune and Neurodegenerative Diseases

Neuroimmune diseases involve dysregulated interactions between the nervous and immune systems, in which persistent immune activation and neuroinflammation can contribute to neurological dysfunction and tissue injury. Gulf War Illness (GWI), for example, has been associated with persistent neuroimmune alterations and chronic neuroinflammation. Neurodegenerative diseases, including Alzheimer’s disease (AD), Parkinson’s disease (PD), Huntington’s disease (HD), and amyotrophic lateral sclerosis (ALS), are characterized by the progressive loss of neuronal structure and function, accompanied by chronic neuroinflammation and the accumulation of pathological protein aggregates. Despite their distinct etiologies and clinical manifestations, neuroimmune and neurodegenerative disorders share chronic neuroinflammation, which may contribute to neuronal dysfunction and disease progression. Elevated levels of TNF-α and IL-6 have been implicated in these processes, highlighting chronic inflammation as a potential mechanistic link between neuroimmune dysfunction and neurodegeneration [97].

Fisetin has been reported to modulate multiple pathways implicated in age-related central nervous system dysfunction and neurodegenerative diseases, including AD, HD, and ALS. Although neuroinflammation represents a shared feature of neuroimmune and neurodegenerative disorders, much of the available mechanistic evidence on fisetin has focused on neurodegenerative disease models. Among these, AD has been particularly well studied in animal models as described below [98,99,100].

Alzheimer’s disease is characterized by amyloid-beta (Aβ) accumulation, synaptic dysfunction, and oxidative stress and currently lacks fully effective therapies. In human SH-SY5Y neuroblastoma cells (differentiated into neuron-like cells), fisetin protected against Aβ_1–42_-induced toxicity by restoring redox balance, enhancing the expression of the antioxidant enzymes superoxide dismutase 1, glutathione reductase, and catalase, and improving mitochondrial function [101]. In preclinical models of AD, fisetin was shown to affect pathways involved in autophagy, as indicated by increased phosphorylation of AMP-activated protein kinase (AMPK) and by increased expression of sirtuin 1 (SIRT1)*,* a key NAD^+^-dependent protein deacetylase that promotes mitochondrial biogenesis and reduces oxidative stress. Additionally, fisetin improved synaptic integrity by increasing the expression of the synaptic markers PSD-95 and SNAP-25 while reducing apoptosis and the accumulation of Aβ and phosphorylated tau [102]. In human neuroblastoma cells, fisetin suppressed several oxidative stress-related genes, including heme oxygenase-1, NAD(P)H dehydrogenase quinone 1, NF-E2-related factor 2, and γ-glutamate-cysteine ligase modifier [103]. Fisetin has been shown to increase mitochondrial biogenesis in SH-SY5Y cells, thereby conferring neuroprotective effects [103], and to prevent apoptosis in human brain microvascular endothelial cells [103,104]. Finally, complementary human evidence identified through our literature search but not included among the registered trials identified through ClinicalTrials.gov or the WHO ICTRP has also been reported. In this study, fisetin was evaluated as an adjunct to recombinant tissue plasminogen activator (rt-PA) in patients with acute ischemic stroke [105]. Fisetin administered as an adjunct to rt-PA improved neurological recovery, particularly when treatment was initiated beyond the conventional therapeutic window. The beneficial effects were associated with attenuation of inflammation and preservation of blood–brain barrier integrity [105]. Collectively, these findings suggest that fisetin may exert neuroprotective effects through multiple mechanisms, including modulation of neuroinflammation, oxidative stress, mitochondrial function, synaptic integrity, and protein homeostasis. However, much of this evidence remains preclinical, highlighting the need to determine whether these effects translate into neurological benefits in humans. The available registered clinical trials investigating fisetin in neuroimmune and neurodegenerative diseases are discussed below.

### Clinical Trials: Neuroimmune and Neurodegenerative Diseases and Fisetin

Among the registered clinical trials identified, 11.8% (4/34) were classified as investigating fisetin effects in neuroimmune or neurodegenerative diseases (Table 2), including Gulf War Illness (NCT02909686), mild cognitive impairment (NCT02741804), primary open-angle glaucoma (NCT04784234), and mild Alzheimer’s disease (NCT07279714). Together, these trials encompass heterogeneous neurological diseases and evaluate fisetin either as an individual intervention or as a component of multimodal formulations and lifestyle interventions.

Of these four trials, two were completed (50%, 2/4), one was terminated (25%, 1/4), and one had an unknown recruitment status (25%, 1/4) (Table 3). However, results were available for only one of the two completed trials (NCT02909686). The completed “Effects of Botanical Microglia Modulators in Gulf War Illness” trial (NCT02909686) evaluated fisetin at doses ranging from 200 to 800 mg/day in individuals with Gulf War Illness. Although the intervention was based on the potential contribution of inflammatory processes to Gulf War Illness, the study primarily evaluated clinical symptoms rather than direct measures of neuroinflammation. Fisetin did not significantly improve symptom severity compared with placebo; however, interpretation is limited by the small number of participants with evaluable fisetin data.

The other completed trial, NCT07279714, evaluated fisetin at 20 mg/kg/day in individuals with mild Alzheimer’s disease, providing a direct clinical investigation of fisetin in a neurodegenerative disease. However, results from this trial were not available at the time of our search, precluding conclusions regarding its efficacy. NCT02741804, which had an unknown recruitment status, evaluates a multimodal lifestyle intervention in individuals with mild cognitive impairment that includes BBH-1001, a multi-component supplement providing fisetin (66.6 mg/day) together with other micronutrients. Consequently, any effects observed in this study cannot necessarily be attributed to fisetin alone. The NCT04784234 trial evaluated GlaucoCetin, another multi-component intervention containing fisetin, in individuals with primary open-angle glaucoma; however, the trial was terminated after the company producing the supplement was acquired and discontinued its production.

Overall, the clinical evidence for fisetin in neuroimmune and neurodegenerative diseases remains limited and heterogeneous. Although two of the four registered trials were completed, results were available for only one, NCT02909686. Its small evaluable sample and lack of significant improvement in clinical symptoms limit conclusions regarding efficacy. Furthermore, two of the remaining studies evaluate fisetin as part of multi-component interventions, complicating attribution of potential effects specifically to fisetin. NCT07279714 is particularly relevant because it directly evaluated fisetin in individuals with mild Alzheimer’s disease; however, in the absence of available results, it remains to be determined whether the neuroprotective effects observed in preclinical models translate into clinically meaningful benefits in individuals with Alzheimer’s disease.

## 10. Other Chronic Age-Related Diseases

Beyond the disease categories discussed above, cellular senescence has been implicated in several chronic age-related diseases characterized by progressive tissue dysfunction, impaired regenerative capacity, and persistent low-grade inflammation. Senescent cells can accumulate in affected tissues and secrete SASP factors that alter the local tissue environment. This mechanism is particularly relevant to musculoskeletal disorders such as osteoarthritis, in which senescent cells accumulate in articular cartilage and other joint tissues and have been implicated in cartilage degradation and disease progression. Given its senolytic and anti-inflammatory properties, fisetin has been investigated as a potential strategy to target senescence-associated mechanisms underlying chronic tissue degeneration.

Preclinical studies provide support for this rationale in osteoarthritis. In particular, in human osteoarthritis chondrocytes, fisetin reduced IL-1β-induced inflammatory and catabolic signaling through activation of SIRT1, while in a mouse model of osteoarthritis, fisetin attenuated cartilage destruction and synovitis [59], suggesting that its potential effects may extend beyond systemic senescent-cell clearance to modulation of the local joint environment. However, these findings remain predominantly preclinical, and whether fisetin can meaningfully alter structural disease progression or improve functional outcomes in humans remains uncertain.

### Clinical Trials: Other Chronic Age-Related Diseases and Fisetin

Among the registered clinical trials identified, 23.5% (8/34) were classified as investigating other chronic age-related diseases (Table 2). These trials predominantly involve musculoskeletal disorders, including knee osteoarthritis (NCT04770064, NCT05482672, NCT04210986, and NCT04815902), meniscus injury and repair (NCT05505747), femoroacetabular impingement (NCT05025956), and carpal tunnel syndrome (NCT05416515). One additional trial investigates CVID-associated interstitial lung disease (NCT05593588), extending the clinical investigation of fisetin beyond musculoskeletal diseases.

Of these eight trials, two were completed (25%, 2/8; NCT05416515 and NCT04210986), three were withdrawn (37.5%, 3/8; NCT04770064, NCT05505747, and NCT05482672), one is active but not recruiting (12.5%, 1/8; NCT05593588), and two have an unknown recruitment status (25%, 2/8; NCT05025956 and NCT04815902). Results were available for both completed trials. NCT04210986 evaluated intermittent fisetin treatment in individuals with knee osteoarthritis, with outcomes including inflammatory and cartilage-degradation biomarkers, physical function, and other clinical parameters. Although results have been reported, the findings do not demonstrate a consistent benefit of fisetin across the clinical and biomarker outcomes evaluated. NCT05416515 evaluated fisetin in 40 individuals with mild-to-moderate carpal tunnel syndrome. The study assessed changes in carpal tunnel symptoms and functional status using the Boston Carpal Tunnel Syndrome Questionnaire, as well as circulating markers of cellular senescence and inflammation, including p16, IL-6, IL-15, TNF, PAI-1, and ICAM-1. Because the study lacked a placebo or untreated control group, any observed changes cannot be attributed definitively to fisetin.

Overall, clinical evidence for fisetin in other chronic age-related diseases remains limited and heterogeneous. Only two of the eight registered trials were completed with results available, while three were withdrawn and two had an unknown recruitment status. Moreover, despite preclinical evidence supporting potential effects of fisetin on senescence, inflammation, and cartilage degeneration in osteoarthritis, the available clinical findings have not yet demonstrated consistent efficacy. These observations highlight the gap between the promising preclinical effects of fisetin and the currently available human evidence and emphasize the need for adequately powered clinical trials with clearly defined clinical and mechanistic outcomes.

## 11. Conclusions

The Geroscience hypothesis posits that therapeutically targeting fundamental mechanisms underlying aging may delay or mitigate age-related diseases [106]. Within this framework, fisetin has emerged as a supplement with potential geroprotective properties, based largely on its senolytic activity in preclinical models of aging and chronic diseases. Preclinical evidence from cellular and animal models suggests that fisetin may modulate cellular senescence, the SASP, inflammation, oxidative stress, and mitochondrial function. Proof-of-concept studies in human cell models and animals support the clinical investigation of fisetin because the mechanisms potentially underlying its effects appear to vary across tissues and disease contexts, extending beyond senescent-cell clearance alone.

The clinical evidence, however, remains considerably less developed than the preclinical literature. The 34 registered trials identified in this review span aging and frailty, metabolic diseases, cancer, CVDs, neuroimmune and neurodegenerative disorders, and other chronic age-related diseases. Several trials also assess outcomes relevant to more than one disease category, reflecting the interconnected nature of aging-related biological processes. Of the 34 registered trials, only 6 were completed, and results were available for 4, representing 11.8% of all identified studies. Many studies remain ongoing, while others have been withdrawn, suspended, terminated, or have an unknown recruitment status. Available human findings are heterogeneous: preliminary studies have reported positive effects on metabolic and vascular outcomes, whereas other completed studies have not demonstrated consistent benefits. Furthermore, interpretation is frequently complicated by small sample sizes, short intervention periods, heterogeneous dosing regimens, differences in study populations and outcomes, and the use of fisetin as part of multimodal interventions, which can prevent clear isolation of its effects.

Available clinical studies have generally used short-term or intermittent dosing regimens, but the current evidence is insufficient to establish the safety of prolonged high-dose supplementation. Safety and tolerability have been assessed in some completed studies and included as outcomes in several ongoing trials, but the available human evidence remains insufficient to establish a long-term safety profile for fisetin. Potential interactions between fisetin and concomitant medications or other therapeutic agents also remain insufficiently characterized. Differences in dose, formulation, treatment duration, and co-interventions further complicate comparisons of safety across trials. Therefore, longer-term studies with systematic adverse-event monitoring, assessment of treatment discontinuation and adherence, and evaluation of potential drug interactions are needed to better define the safety profile of fisetin in humans.

These limitations highlight the importance of establishing clinical efficacy. Future studies should prioritize adequately powered, randomized, placebo-controlled trials and clearly distinguish the effects of fisetin alone from those of combination interventions. Attention is also needed to dose selection, treatment schedules, and formulation. Clinical outcomes should be accompanied by biomarkers of cellular senescence, and longer follow-up will be particularly relevant for determining fisetin safety.

Overall, the available evidence supports continued clinical investigation of fisetin but does not yet establish its effectiveness as a geroprotective therapy in humans. Results from ongoing and recently completed clinical trials will be important for determining whether the effects of fisetin observed in preclinical studies translate into clinically meaningful benefits in humans.

## Figures and Tables

**Table 1:** Structured search strategies used across databases and clinical trial registries to identify studies evaluating the role of fisetin in age-related diseases. We searched four bibliographic databases (PubMed/MEDLINE, Scopus, Web of Science [WoS], and Google Scholar) to identify literature supporting the narrative discussion, including mechanistic, translational, and contextual evidence. We searched in two clinical trial registries (ClinicalTrials.gov and the WHO International Clinical Trials Registry Platform [ICTRP]) to identify registered human clinical trials for the structured descriptive synthesis.

Source	Date Searched	Search Strategy	Filters/Limits	Records Identified
ClinicalTrials.gov	14 August 2026	Disease: Chronic disease OR chronic low-grade inflammatory disease OR inflammation OR inflammatory disease OR aging OR healthy aging OR frailty OR cancer OR neuroimmune disease OR neurodegenerative disease OR cognitive impairment OR glaucoma OR metabolic disease OR obesity OR diabetes OR cardiovascular disease OR osteoarthritis OR knee osteoarthritis OR skeletal health OR interstitial lung disease OR carpal tunnel syndrome OR femoroacetabular impingement OR meniscus Intervention/treatment: Fisetin OR Fisetin-fed condition OR Fisetin intervention OR Fisetin dose		32
WHO ICTRP	14 August 2026	In the title: Chronic disease OR chronic low-grade inflammatory disease OR inflammation OR inflammatory disease OR aging OR healthy aging OR frailty OR cancer OR neuroimmune disease OR neurodegenerative disease OR cognitive impairment OR glaucoma OR metabolic disease OR obesity OR diabetes OR cardiovascular disease OR osteoarthritis OR knee osteoarthritis OR skeletal health OR interstitial lung disease OR carpal tunnel syndrome OR femoroacetabular impingement OR meniscus In the condition: Chronic disease OR chronic low-grade inflammatory disease OR inflammation OR inflammatory disease OR aging OR healthy aging OR frailty OR cancer OR neuroimmune disease OR neurodegenerative disease OR cognitive impairment OR glaucoma OR metabolic disease OR obesity OR diabetes OR cardiovascular disease OR osteoarthritis OR knee osteoarthritis OR skeletal health OR interstitial lung disease OR carpal tunnel syndrome OR femoroacetabular impingement OR meniscus In the intervention: Fisetin OR Fisetin-fed condition OR Fisetin intervention OR Fisetin dose	Recruitment status is: ALL	18
PubMed/MEDLINE	14 August 2026	(Chronic disease OR chronic low-grade inflammatory disease OR inflammation OR inflammatory disease OR aging OR healthy aging OR frailty OR cancer OR neuroimmune disease OR neurodegenerative disease OR cognitive impairment OR glaucoma OR metabolic disease OR obesity OR diabetes OR cardiovascular disease OR osteoarthritis OR knee osteoarthritis OR skeletal health OR interstitial lung disease OR carpal tunnel syndrome OR femoroacetabular impingement OR meniscus AND (fisetin) AND (“Humans” [MeSH Terms])	NOT review [Publication Type] NOT meta-analysis [Publication Type] AND English [Language]	420
Scopus	14 August 2026	TITLE-ABS-KEY(“chronic disease” OR “chronic low-grade inflammatory disease” OR inflammation OR “inflammatory disease” OR aging OR “healthy aging” OR frailty OR cancer OR “neuroimmune disease” OR “neurodegenerative disease” OR “cognitive impairment” OR glaucoma OR “metabolic disease” OR obesity OR diabetes OR “cardiovascular disease” OR osteoarthritis OR “knee osteoarthritis” OR “skeletal health” OR “interstitial lung disease” OR “carpal tunnel syndrome” OR “femoroacetabular impingement” OR meniscus) AND TITLE-ABS-KEY(fisetin)	LIMIT-TO(LANGUAGE, “English”) AND LIMIT-TO(DOCTYPE, “ar”) AND NOT TITLE-ABS-KEY(mice OR mouse OR murine OR rats OR rat OR rodents OR rodent OR “animal model” OR “animal models” OR “in vitro” OR “cell culture” OR “cell line” OR “cell lines”)	283
Web of Science (WoS)	14 August 2026	TS = (“chronic disease” OR “chronic low-grade inflammatory disease” OR inflammation OR “inflammatory disease” OR aging OR “healthy aging” OR frailty OR cancer OR “neuroimmune disease” OR “neurodegenerative disease” OR “cognitive impairment” OR glaucoma OR “metabolic disease” OR obesity OR diabetes OR “cardiovascular disease” OR osteoarthritis OR “knee osteoarthritis” OR “skeletal health” OR “interstitial lung disease” OR “carpal tunnel syndrome” OR “femoroacetabular impingement” OR meniscus) AND TS = (fisetin)	AND LA = (English) AND DT = (Article) NOT TS = (mouse OR mice OR murine OR rat OR rats OR rodent OR rodents OR “animal model” OR “animal models” OR “in vitro” OR “cell culture” OR “cell cultures” OR “cell line” OR “cell lines”)	291
Google Scholar	14 August 2026	Find articles with all of the words: Fisetin, human with at least one of the words: patient, patients, participant, participants, subjects, clinical trial, intervention, treatment, supplementation, aging, frailty, cancer, inflammation, osteoarthritis, diabetes, cardiovascular, pharmacokinetic	without the words: mouse, mice, murine, rat, rats, rodent, rodents, review, meta-analysis, cell line, COVID. Limit language: English. Include citations	51

## Data Availability

No new data were created or analyzed in this study. Data sharing is not applicable to this article.

## Abbreviations

The following abbreviations are used in this manuscript:

Abbreviation	Meaning
AD	Alzheimer’s disease
ALS	Amyotrophic lateral sclerosis
AMPK	AMP-activated protein kinase
AUC	Area under the curve
BC	Body composition
BMAC	Bone marrow aspirate concentrate
BMI	Body mass index
C/EBPα	CCAAT/enhancer-binding protein alpha
C12FDG	5-Dodecanoylaminofluorescein di-β-d-galactopyranoside
CHD	Coronary heart disease
CNS	Central nervous system
COX-2	Cyclooxygenase-2
CRP	C-reactive protein
CTX	C-terminal telopeptide of type I collagen
CVDs	Cardiovascular diseases
CXCL12	C-X-C motif chemokine ligand 12
DNA	Deoxyribonucleic acid
DXA	Dual-energy X-ray absorptiometry
ERK1/2	Extracellular signal-regulated kinase 1/2
FABP4	Fatty acid-binding protein 4
GRECC	Geriatric Research, Education and Clinical Center
H_2_O_2_	Hydrogen peroxide
HD	Huntington’s disease
HO-1	Heme oxygenase-1
HPFS	Health Professionals Follow-up Study
ICAM-1	Intercellular adhesion molecule-1
ICTRP	International Clinical Trials Registry Platform
IL	Interleukin
IL-1β	Interleukin-1 beta
IL-4	Interleukin-4
IL-6	Interleukin-6
IL-8	Interleukin-8
IL-15	Interleukin-15
LDL	Low-density lipoprotein
MAPK	Mitogen-activated protein kinase
MFN2	Mitofusin-2
MK2	MAPK-activated protein kinase 2
MMP-9	Matrix metalloproteinase-9
mTOR	Mammalian (mechanistic) target of rapamycin
mTORC1	mTOR complex 1
mTORC2	mTOR complex 2
NAD^+^	Nicotinamide adenine dinucleotide (oxidized form)
NF-κB	Nuclear factor kappa B
NF-E2	Nuclear factor erythroid 2
NHS	Nurses’ Health Study
NO	Nitric oxide
NR	Not reported
Nrf2	Nuclear factor erythroid 2-related factor 2
P1NP	Procollagen type I N-terminal propeptide
PAD	Peripheral artery disease
PAI-1	Plasminogen activator inhibitor-1
PD	Parkinson’s disease
PI3K	Phosphoinositide 3-kinase
PPARγ	Peroxisome proliferator-activated receptor gamma
PSD-95	Postsynaptic density protein 95
PTEN	Phosphatase and tensin homolog
RAGE	Receptor for advanced glycation end products
ROS	Reactive oxygen species
rt-PA	Recombinant tissue plasminogen activator
SASP	Senescence-associated secretory phenotype
SIRT1	Sirtuin 1
SNAP-25	Synaptosomal-associated protein 25
STAT3	Signal transducer and activator of transcription 3
S6K	Ribosomal protein S6 kinase
T2DM	Type 2 diabetes mellitus
TNF-α	Tumor necrosis factor alpha
VEGF	Vascular endothelial growth factor
WoS	Web of Science
3T3-L1	Mouse preadipocyte cell line
A431	Human epidermoid carcinoma cell line
ADSCs	Adipose-derived stem cells
C2C12	Mouse myoblast cell line
FLS	Fibroblast-like synoviocytes
HeLa	Human cervical cancer cell line
HUVECs	Human umbilical vein endothelial cells
IMR90	Human lung fibroblast cell line
MCF-7	Human breast cancer cell line
MSCs	Mesenchymal stem/stromal cells
NSCLC	Non-small cell lung cancer cells
PBMCs	Peripheral blood mononuclear cells
PC3	Human prostate cancer cell line
SH-SY5Y	Human neuroblastoma cell line
VSMCs	Vascular smooth muscle cells
